# Supplementary material for: Impact of early nutrition and feeding route on clinical outcomes of neurocritically ill patients
Source: PLoS One. 2023 Mar 23;18(3):e0283593. doi: 10.1371/journal.pone.0283593 (PMC10035931; doi:10.1371/journal.pone.0283593)
Supplement: S1 Table — (DOCX) [file pone.0283593.s001.docx]

**Supplementary table 1.** Baseline characteristics according to timing of nutrition in the overall, PSM and PSOW adjusted population

|  | **Overall** | | | | **PSM** | | | | **PSOW** | | | |
| --- | --- | --- | --- | --- | --- | --- | --- | --- | --- | --- | --- | --- |
|  | **Late nutrition** | **Early nutrition** | ***p*** | **SMD** | **Late nutrition** | **Early nutrition** | ***p*** | **SMD** | **Late nutrition** | **Early nutrition** | ***p*** | **SMD** |
| ***n*** | ***969*** | ***384*** |  |  | ***377*** | ***377*** |  |  | ***252.8*** | ***252.8*** |  |  |
| **Patient demographics** |  |  |  |  |  |  |  |  |  |  |  |  |
| **Age (year)** | 50.1 (23.7) | 51.7 (22.1) | 0.267 | 0.068 | 50.7 (22.4) | 51.2 (22.0) | 0.764 | 0.022 | 50.7 (23.2) | 50.7 (22.4) | 1 | <0.001 |
| **Sex, male** | 512 (52.8) | 195 (50.8) | 0.534 | 0.041 | 181 (48.0) | 192 (50.9) | 0.466 | 0.058 | 131.0 (51.8) | 131.0 (51.8) | 1 | <0.001 |
| **Comorbidities** |  |  |  |  |  |  |  |  |  |  |  |  |
| **Malignancy** | 528 (54.5) | 220 (57.3) | 0.382 | 0.056 | 218 (57.8) | 219 (58.1) | 1 | 0.005 | 146.6 (58.0) | 146.6 (58.0) | 1 | <0.001 |
| **Hypertension** | 332 (34.3) | 135 (35.2) | 0.804 | 0.019 | 137 (36.3) | 131 (34.7) | 0.704 | 0.033 | 88.9 (35.2) | 88.9 (35.2) | 1 | <0.001 |
| **Diabetes mellitus** | 131 (13.5) | 58 (15.1) | 0.502 | 0.045 | 52 (13.8) | 57 (15.1) | 0.679 | 0.038 | 36.9 (14.6) | 36.9 (14.6) | 1 | <0.001 |
| **Chronic kidney disease** | 64 (6.6) | 32 (8.3) | 0.318 | 0.066 | 32 (8.5) | 30 (8.0) | 0.895 | 0.019 | 19.4 (7.7) | 19.4 (7.7) | 1 | <0.001 |
| **Cardiovascular disease** | 42 (4.3) | 10 (2.6) | 0.182 | 0.095 | 8 (2.1) | 10 (2.7) | 0.811 | 0.035 | 8.0 (3.2) | 8.0 (3.2) | 1 | <0.001 |
| **Chronic liver disease** | 30 (3.1) | 16 (4.2) | 0.416 | 0.057 | 19 (5.0) | 16 (4.2) | 0.729 | 0.038 | 9.5 (3.8) | 9.5 (3.8) | 1 | <0.001 |
| **Behavioral risk factors** |  |  |  |  |  |  |  |  |  |  |  |  |
| **Current alcohol consumption** | 199 (20.5) | 86 (22.4) | 0.495 | 0.045 | 94 (24.9) | 85 (22.5) | 0.494 | 0.056 | 56.0 (22.2) | 56.0 (22.2) | 1 | <0.001 |
| **Current smoking** | 98 (10.1) | 52 (13.5) | 0.086 | 0.106 | 48 (12.7) | 51 (13.5) | 0.829 | 0.024 | 30.8 (12.2) | 30.8 (12.2) | 1 | <0.001 |
| **Cause of ICU admission** |  |  | 0.002 | 0.295 |  |  | 0.933 | 0.127 |  |  | 1 | <0.001 |
| **Brain tumor** | 351 (36.2) | 157 (40.9) |  |  | 158 (41.9) | 155 (41.1) |  |  | 103.5 (41.0) | 103.5 (41.0) |  |  |
| **Elective vascular surgery** | 72 (7.4) | 37 (9.6) |  |  | 29 (7.7) | 37 (9.8) |  |  | 23.6 (9.3) | 23.6 (9.3) |  |  |
| **Intracerebral hemorrhage** | 179 (18.5) | 56 (14.6) |  |  | 51 (13.5) | 56 (14.9) |  |  | 38.8 (15.4) | 38.8 (15.4) |  |  |
| **Traumatic brain injury** | 152 (15.7) | 39 (10.2) |  |  | 45 (11.9) | 39 (10.3) |  |  | 29.6 (11.7) | 29.6 (11.7) |  |  |
| **Subarachnoid hemorrhage** | 122 (12.6) | 50 (13.0) |  |  | 58 (15.4) | 50 (13.3) |  |  | 32.4 (12.8) | 32.4 (12.8) |  |  |
| **Spinal surgery** | 17 (1.8) | 12 (3.1) |  |  | 10 (2.7) | 10 (2.7) |  |  | 6.5 (2.6) | 6.5 (2.6) |  |  |
| **Central nervous system infection** | 12 (1.2) | 13 (3.4) |  |  | 11 (2.9) | 10 (2.7) |  |  | 5.9 (2.3) | 5.9 (2.3) |  |  |
| **Cerebral infarction** | 22 (2.3) | 11 (2.9) |  |  | 8 (2.1) | 11 (2.9) |  |  | 6.6 (2.6) | 6.6 (2.6) |  |  |
| **Others** | 42 (4.3) | 9 (2.3) |  |  | 7 (1.9) | 9 (2.4) |  |  | 5.9 (2.3) | 5.9 (2.3) |  |  |
| **APACHE II score on ICU admission** | 8.31 (7.7) | 6.13 (5.5) | <0.001 | 0.326 | 6.2 (6.0) | 6.2 (5.5) | 0.89 | 0.01 | 6.4 (6.4) | 6.4 (5.8) | 1 | <0.001 |
| **Glasgow coma scale on ICU admission** | 11.8 (4.4) | 13.7 (2.5) | <0.001 | 0.549 | 13.7 (2.7) | 13.7 (2.5) | 0.91 | 0.008 | 13.4 (3.0) | 13.4 (2.8) | 1 | <0.001 |
| **ICU management** |  |  |  |  |  |  |  |  |  |  |  |  |
| **Use of vasopressors** | 160 (16.5) | 45 (11.7) | 0.033 | 0.138 | 44 (11.7) | 45 (11.9) | 1 | 0.008 | 31.7 (12.5) | 31.7 (12.5) | 1 | <0.001 |
| **Mechanical ventilation** | 652 (67.3) | 207 (53.9) | <0.001 | 0.276 | 211 (56.0) | 206 (54.6) | 0.77 | 0.027 | 145.0 (57.4) | 145.0 (57.4) | 1 | <0.001 |
| **Continuous renal replacement therapy** | 39 (4.0) | 10 (2.6) | 0.272 | 0.079 | 10 (2.7) | 10 (2.7) | 1 | <0.001 | 7.2 (2.8) | 7.2 (2.8) | 1 | <0.001 |
| **ICP monitoring** | 407 (42.0) | 182 (47.4) | 0.081 | 0.109 | 165 (43.8) | 179 (47.5) | 0.342 | 0.075 | 118.7 (46.9) | 118.7 (46.9) | 1 | <0.001 |
| **Use of mannitol*** | 406 (41.9) | 167 (43.5) | 0.636 | 0.032 | 160 (42.4) | 161 (42.7) | 1 | 0.005 | 107.6 (42.6) | 107.6 (42.6) | 1 | <0.001 |
| **Use of glycerin*** | 391 (40.4) | 151 (39.3) | 0.775 | 0.021 | 161 (42.7) | 151 (40.1) | 0.506 | 0.054 | 102.8 (40.7) | 102.8 (40.7) | 1 | <0.001 |
| **Clinical outcomes†** |  |  |  |  |  |  |  |  |  |  |  |  |
| **In-hospital mortality** | 321 (33.1) | 54 (14.1) | <0.001 |  | 67 (17.6) | 54 (14.2) | 0.234 |  | 51.7 (20.5) | 41.1 (16.2) | 0.094 |  |
| **28-day mortality** | 295 (30.4) | 46 (12.0) | <0.001 |  | 54 (14.2) | 46 (12.1) | 0.453 |  | 45.1 (17.8) | 35.3 (14.0) | 0.105 |  |
| **ICU mortality** | 281 (29.0) | 38 (9.9) | <0.001 |  | 47 (12.3) | 38 (10.0) | 0.357 |  | 40.4 (16.0) | 29.8 (11.8) | 0.065 |  |
| **ICU length of stay (hour)** | 68.9 (253.3) | 78.2 (177.9) | 0.511 |  | 89.4 (281.5) | 78.1 (178.6) | 0.508 |  | 75.7 (227.4) | 78.8 (187.3) | 0.806 |  |
| **Hospital length of stay (day)** | 292.1 (769.3) | 329.7 (989.9) | 0.457 |  | 322.7 (448.1) | 330.5 (993.7) | 0.888 |  | 318.5 (782.4) | 342.4 (1089.8) | 0.719 |  |
| **Infectious complications** | 82 (8.5) | 32 (8.3) | 1 |  | 46 (12.1) | 32 (8.4) | 0.12 |  | 318.5 (782.4) | 342.4 (1089.8) | 0.297 |  |

Data are presented as numbers (%) or means ± standard deviations.

*Some patients received more than one hyperosmolar agent.

†Variables are not retained in propensity score matching.

PSM, propensity score matching; PSOW, propensity score weighting using overlap weights.

APACHE II, Acute Physiology and Chronic Health Evaluation; ICP, intracranial pressure, ICU, intensive care unit; SMD, standardized mean difference.
