# Supplementary material for: Impact of early nutrition and feeding route on clinical outcomes of neurocritically ill patients
Source: PLoS One. 2023 Mar 23;18(3):e0283593. doi: 10.1371/journal.pone.0283593 (PMC10035931; doi:10.1371/journal.pone.0283593)
Supplement: S2 Table — (DOCX) [file pone.0283593.s002.docx]

**Supplementary table 2.** Baseline characteristics of patients with and without early enteral feeding in the overall, PSM and PSOW adjusted population.

|  | **Overall** | | | | **PSM** | | | | **PSOW** | | | |
| --- | --- | --- | --- | --- | --- | --- | --- | --- | --- | --- | --- | --- |
|  | **No-EEN (late nutrition)** | **EEN** | ***p*** | **SMD** | **No-EEN (late nutrition)** | **EEN** | ***p*** | **SMD** | **No-EEN (late nutrition)** | **EEN** | ***p*** | **SMD** |
| ***n*** | ***969*** | ***152*** |  |  | ***149*** | ***149*** |  |  | ***115*** | ***115*** |  |  |
| **Patient demographics** |  |  |  |  |  |  |  |  |  |  |  |  |
| **Age (year)** | 50.1 (23.7) | 44.0 (25.7) | 0.004 | 0.247 | 43.2 (26.1) | 43.7 (25.8) | 0.85 | 0.022 | 44.0 (25.9) | 44.0 (25.7) | 1 | <0.001 |
| **Sex, male** | 512 (52.8) | 75 (49.3) | 0.475 | 0.07 | 77 (51.7) | 72 (48.3) | 0.643 | 0.067 | 58.1 (50.5) | 58.1 (50.5) | 1 | <0.001 |
| **Comorbidities** |  |  |  |  |  |  |  |  |  |  |  |  |
| **Malignancy** | 528 (54.5) | 98 (64.5) | 0.027 | 0.204 | 94 (63.1) | 97 (65.1) | 0.809 | 0.042 | 74.8 (65.1) | 74.8 (65.1) | 1 | <0.001 |
| **Hypertension** | 332 (34.3) | 45 (29.6) | 0.299 | 0.1 | 43 (28.9) | 44 (29.5) | 1 | 0.015 | 33.8 (29.4) | 33.8 (29.4) | 1 | <0.001 |
| **Diabetes mellitus** | 131 (13.5) | 14 (9.2) | 0.18 | 0.136 | 14 (9.4) | 14 (9.4) | 1 | <0.001 | 10.5 (9.1) | 10.5 (9.1) | 1 | <0.001 |
| **Chronic kidney disease** | 64 (6.6) | 13 (8.6) | 0.478 | 0.074 | 15 (10.1) | 12 (8.1) | 0.686 | 0.07 | 9.1 (7.9) | 9.1 (7.9) | 1 | <0.001 |
| **Cardiovascular disease** | 42 (4.3) | 4 (2.6) | 0.445 | 0.093 | 4 (2.7) | 4 (2.7) | 1 | <0.001 | 3.4 (3.0) | 3.4 (3.0) | 1 | <0.001 |
| **Chronic liver disease** | 30 (3.1) | 8 (5.3) | 0.258 | 0.108 | 5 (3.4) | 8 (5.4) | 0.571 | 0.099 | 4.8 (4.2) | 4.8 (4.2) | 1 | <0.001 |
| **Behavioral risk factors** |  |  |  |  |  |  |  |  |  |  |  |  |
| **Current alcohol consumption** | 199 (20.5) | 23 (15.1) | 0.148 | 0.142 | 27 (18.1) | 23 (15.4) | 0.642 | 0.072 | 18.3 (15.9) | 18.3 (15.9) | 1 | <0.001 |
| **Current smoking** | 98 (10.1) | 13 (8.6) | 0.651 | 0.054 | 15 (10.1) | 12 (8.1) | 0.686 | 0.07 | 10.0 (8.7) | 10.0 (8.7) | 1 | <0.001 |
| **Cause of ICU admission** |  |  | <0.001 | 0.642 |  |  | 0.991 | 0.147 |  |  | 1 | <0.001 |
| **Brain tumor** | 351 (36.2) | 72 (47.4) |  |  | 67 (45.0) | 72 (48.3) |  |  | 55.8 (48.5) | 55.8 (48.5) |  |  |
| **Elective vascular surgery** | 72 (7.4) | 23 (15.1) |  |  | 22 (14.8) | 22 (14.8) |  |  | 16.4 (14.2) | 16.4 (14.2) |  |  |
| **Intracerebral hemorrhage** | 179 (18.5) | 14 (9.2) |  |  | 15 (10.1) | 14 (9.4) |  |  | 11.9 (10.4) | 11.9 (10.4) |  |  |
| **Traumatic brain injury** | 152 (15.7) | 5 (3.3) |  |  | 8 (5.4) | 5 (3.4) |  |  | 4.7 (4.1) | 4.7 (4.1) |  |  |
| **Subarachnoid hemorrhage** | 122 (12.6) | 22 (14.5) |  |  | 20 (13.4) | 21 (14.1) |  |  | 15.6 (13.6) | 15.6 (13.6) |  |  |
| **Spinal surgery** | 17 (1.8) | 4 (2.6) |  |  | 5 (3.4) | 4 (2.7) |  |  | 2.9 (2.5) | 2.9 (2.5) |  |  |
| **Central nervous system infection** | 12 (1.2) | 7 (4.6) |  |  | 7 (4.7) | 6 (4.0) |  |  | 3.7 (3.2) | 3.7 (3.2) |  |  |
| **Cerebral infarction** | 22 (2.3) | 2 (1.3) |  |  | 1 (0.7) | 2 (1.3) |  |  | 1.7 (1.5) | 1.7 (1.5) |  |  |
| **Others** | 42 (4.3) | 3 (2.0) |  |  | 4 (2.7) | 3 (2.0) |  |  | 2.3 (2.0) | 2.3 (2.0) |  |  |
| **APACHE II score on ICU admission** | 8.3 (7.7) | 5.8 (4.4) | <0.001 | 0.404 | 5.8 (5.4) | 5.8 (4.5) | 0.963 | 0.005 | 5.7 (5.5) | 5.7 (4.5) | 1 | <0.001 |
| **Glasgow coma scale on ICU admission** | 11.8 (4.4) | 14.5 (1.3) | <0.001 | 0.849 | 14.6 (1.3) | 14.5 (1.3) | 0.591 | 0.062 | 14.4 (1.7) | 14.4 (1.4) | 1 | <0.001 |
| **ICU management** |  |  |  |  |  |  |  |  |  |  |  |  |
| **Use of vasopressors** | 160 (16.5) | 16 (10.5) | 0.077 | 0.176 | 20 (13.4) | 16 (10.7) | 0.594 | 0.082 | 12.9 (11.2) | 12.9 (11.2) | 1 | <0.001 |
| **Mechanical ventilation** | 652 (67.3) | 52 (34.2) | <0.001 | 0.701 | 57 (38.3) | 52 (34.9) | 0.63 | 0.07 | 44.3 (38.5) | 44.3 (38.5) | 1 | <0.001 |
| **Continuous renal replacement therapy** | 39 (4.0) | 1 (0.7) | 0.065 | 0.224 | 2 (1.3) | 1 (0.7) | 1 | 0.067 | 0.8 (0.7) | 0.8 (0.7) | 1 | <0.001 |
| **ICP monitoring** | 407 (42.0) | 77 (50.7) | 0.055 | 0.174 | 73 (49.0) | 77 (51.7) | 0.728 | 0.054 | 57.9 (50.3) | 57.9 (50.3) | 1 | <0.001 |
| **Use of mannitol*** | 406 (41.9) | 74 (48.7) | 0.138 | 0.137 | 72 (48.3) | 72 (48.3) | 1 | <0.001 | 54.8 (47.6) | 54.8 (47.6) | 1 | <0.001 |
| **Use of glycerin*** | 391 (40.4) | 32 (21.1) | <0.001 | 0.428 | 26 (17.4) | 32 (21.5) | 0.464 | 0.102 | 27.7 (24.0) | 27.7 (24.0) | 1 | <0.001 |
| **Clinical outcomes†** |  |  |  |  |  |  |  |  |  |  |  |  |
| **In-hospital mortality** | 321 (33.1) | 9 (5.9) | <0.001 |  | 19 (12.8) | 9 (6.0) | 0.074 |  | 13.7 (11.9) | 7.7 (6.7) | 0.078 |  |
| **28-day mortality** | 295 (30.4) | 8 (5.3) | <0.001 |  | 15 (10.1) | 8 (5.4) | 0.193 |  | 11.1 (9.7) | 6.3 (5.5) | 0.114 |  |
| **ICU mortality** | 281 (29.0) | 5 (3.3) | <0.001 |  | 15 (10.1) | 5 (3.4) | 0.037 |  | 9.3 (8.1) | 4.1 (3.5) | 0.059 |  |
| **ICU length of stay (hour)** | 292.1 (769.3) | 377.8 (1540.6) | 0.282 |  | 320.8 (498.1) | 381.7 (1555.9) | 0.649 |  | 312.4 (760.8) | 407.1 (1675.6) | 0.527 |  |
| **Hospital length of stay (day)** | 68.9 (253.3) | 95.2 (263.7) | 0.237 |  | 86.6 (221.4) | 96.1 (266.3) | 0.736 |  | 78.1 (218.4) | 103.4 (285.5) | 0.35 |  |
| **Infectious complications** | 82 (8.5) | 3 (2.0) | 0.008 |  | 16 (10.7) | 3 (2.0) | 0.004 |  | 10.0 (8.7) | 2.4 (2.1) | 0.007 |  |

Data are presented as numbers (%) or means ± standard deviations.

*Some patients received more than one hyperosmolar agent.

†Variables are not retained in propensity score matching.

PSM, propensity score matching; PSOW, propensity score weighting using overlap weights.

EEN, early enteral nutrition; APACHE II, Acute Physiology and Chronic Health Evaluation; ICP, intracranial pressure, ICU, intensive care unit; SMD, standardized mean difference.
