# Supplementary material for: Impact of early nutrition and feeding route on clinical outcomes of neurocritically ill patients
Source: PLoS One. 2023 Mar 23;18(3):e0283593. doi: 10.1371/journal.pone.0283593 (PMC10035931; doi:10.1371/journal.pone.0283593)
Supplement: Attachment — Submitted filename: Response to reviewers PLoS ONE_final.docx [file pone.0283593.s003.docx]

July 11 2022

Dr. Rishabh Charan Choudhary

PLOS ONE

Manuscript ID: PONE-D-21-39517

Title: Impact of early nutrition and feeding route on clinical outcomes of neurocritically ill patients

Dear Dr. Rishabh Charan Choudhary

Thank you very much for your letter and for the helpful comment from the reviewer. We appreciate the opportunity to resubmit our revised manuscript entitled **“Impact of early nutrition and feeding route on clinical outcomes of neurocritically ill patients”**. As always, you and your editorial staff have again provided us with a comprehensive and prompt review. Many of the valuable and constructive points that the reviewers pointed out were well taken by all the authors. After going over the reviewer’s comments, my colleagues and I have performed additional investigation and made some revisions in hopes of improving our paper. The revised and added portions of the manuscript are stated in the “Response to Reviewers” and are underlined and highlighted in the revised manuscript for your convenience.

All authors contributed to the conception and interpretation of data, drafting of the manuscript, revising it critically for important intellectual content, and final approval of the manuscript. The whole manuscript or part of it, neither has been published and is not being considered for publication elsewhere in any language except as an abstract. None of the authors have any financial relationships with any company or any other bias or conflict of interest.

We believe that these findings have scientific and clinical impact and will be interesting and informative to your readers. We hope that, upon review, our study will be found to be meritorious of publication in the PLOS ONE.

Yours sincerely,

Jeong-Am Ryu, M.D., Ph.D.

Department of Critical Care Medicine and Department of Neurosurgery, Samsung Medical Center, Sungkyunkwan University School of Medicine, 81 Irwon-ro, Gangnam-gu, Seoul 06351, Republic of Korea

Tel: 82-2-3410-6399, Fax: 82-2-2148-7088

E-mail: lamyud.ryu@samsung.com

**Response to Reviewers**

**Reviewer #1:**

**Comments: The authors have provided a very thorough analysis of a retrospective dataset and have added to the body of literature on this important subject. They have also taken into consideration the limitations of this type of study.**

**Concerns:**

**In Table 2, it is not clear what the p-values indicate. The legend should indicate what comparison is being evaluated. Is it the comparison between the EEN and the Non-EEN patients or the comparison between the EEN and the EPN patients or between the early nutrition and the late nutrition patients? Based on the title of the table, I would presume that it is the comparison between the EEN and the Non-EEN patients.**

R. We apologize for the lack of clear statement. We compared late nutrition, EPN and EEN. We wanted to show which of the late nutrition, EPN, and EEN was the better form of nutrition. As your recommendation, we added the following legend indicated what comparison to revised table 2.

Data show a comparison between late nutrition, EPN and EEN.

**In general, the sample size for this study is rather small for the number of variables considered in the analysis. As the authors noted, the number of patients with EEN and with EPN were small and the severity of illness is notably different between these two groups and the late nutrition group of patients.**

R. We agree with the reviewer’s comment. There were differences in the number of subjects and the severity of illness among the nutrition groups. In this study, there were small sample size of subjects with EEN or EPN. In addition, it would be difficult to provide appropriate nutrition to neurocritically ill patients in the early stage. Nutritional support may be often ignored in early stages of patients with severely injured brain. Therefore, it is difficult to evaluate association between early proper nutrition and clinical outcomes of neurocritically ill patients. As a result, we performed various statistical methods to correct the bias. We evaluated whether early nutrition *per se* was associated with poor prognosis when severity and factors other than nutritional support were controlled by propensity score matching and propensity score weighting.

**Other investigators have found that mortality was higher in critically ill children who received parenteral nutrition or that late parenteral nutrition was associated with better outcomes than early parenteral nutrition in critically ill children. Thus, combining the EEN and the EPN groups might not be beneficial. In fact, including the EPN group with the late nutrition group might not be beneficial, particularly for such a small number of patients.**

R. We agree with the reviewer’s comment. Late parenteral nutrition may be associated with faster recovery and fewer complications, as compared with EPN (Ref. 21). Therefore, combining the EEN and the EPN groups, or the late nutrition and the EPN group might not be beneficial. EPN might affect the outcome as a confounding factor. As your recommendation, we re-analyzed the difference between EEN and non-EEN (only late nutrition, except EPN). We revised Figure 2 & 3 and added the following sentences in the Discussion section of the revised manuscript (line 214-218 in page 15).

EPN may be associated with delayed recovery and more complications, as compared with late parenteral nutrition (Ref. 21). Therefore, combining the EEN group with the EPN group, or the late nutrition group with the EPN group might not be useful in evaluating the association between feeding options and clinical outcomes. EPN might affect the outcome as a confounding factor in this study.

Ref. 21: Casaer MP, Mesotten D, Hermans G, Wouters PJ, Schetz M, Meyfroidt G, et al. Early versus late parenteral nutrition in critically ill adults. N Engl J Med. 2011; 365: 506-17. https://doi.org/10.1056/NEJMoa1102662 PMID: 21714640

**Other factors which should be considered would be the amount of nutrition provided to patients with respect to caloric intake and protein intake. The timing of nutrition may not be as important as the quantity and the composition of the nutrition provided. However, even the timing may impact outcomes. Why did the investigators choose to define early nutrition to be any nutrition before 72 hours? Did they consider 48 hours or even 24 hours?**

R. We agree with the reviewer’s comment. In Dr. Wang’s study (Ref. 8), which was a well-designed meta-analysis, early nutrition was defined as 72 hours. In our study, early nutrition was also defined as feeding within 72 hours referring to the Dr. Wang’s study. Even the early nutrition, which was defined as feeding within 72 hours, had a smaller sample size in our study. Therefore, it was difficult to perform a statistical analysis with the definition of feeding within 24 or 48 hours because the sample sizes would be so small in these definitions. As a result, in this study, early nutrition was defined as feeding within 72 hours referring to the Dr. Wang’s study.

Ref. 8: Wang X, Dong Y, Han X, Qi XQ, Huang CG, Hou LJ. Nutritional support for patients sustaining traumatic brain injury: a systematic review and meta-analysis of prospective studies. PLoS One. 2013; 8: e58838. https://doi.org/10.1371/journal.pone.0058838 PMID: 23527035

In addition, there was the limitation of this study. Unfortunately, the amount of EEN or EPN calorie intake for patients in the early stage was not considered in this study due to its retrospective nature. We have mentioned these limitations in the limitation section of the revised manuscript.

The amount of EEN or EPN calorie intake for patients in the early stage was not considered in this study due to its retrospective nature.

**Finally, I believe the investigators provided the various methods of data analysis to demonstrate a thorough approach, which is impressive, but presenting all analyses is probably unnecessary. Which analytic method is the best for this type of study? Which method retains the most data and most appropriately fits this study design and addresses the concerns of selection bias and confounding by comorbidities, clinical diagnoses, and severity of illness. This should have been determined in advance.**

R. We agree with the reviewer’s comment. We performed various statistical methods to correct the bias. However, as your concerns, various statistical methods can confuse the readers. PSTW (a relatively rare method) and IPTW (an inappropriate balance, frequently over 0.1 of SMD) has been removed from the original manuscript. As following graph, severity of the subjects and factors other than nutritional support were well controlled by PSM and PSOW in this study. We revised Figure 2 & 3.

**I would recommend revising the analysis to compare the quantity and the composition of the nutrition provided and I would consider excluding those who received EPN from the comparison of EEN to non-EEN. I would also limit the different types of analyses to reflect the most appropriate use of the data.**

R. We agree with the reviewer’s comment. As reviewer’s recommendation, those who received EPN have been excluded from the comparison of EEN to non-EEN. We re-analyzed the difference between EEN and non-EEN (only late nutrition, except EPN). We revised Table 2. In addition, PSTW and IPTW has been removed from the original manuscript because of a relatively rare method or an inappropriate balance.

We thank the reviewer for valuable comments. Addressing them fully has significantly strengthened the manuscript.

**Reviewer #2:**

**Dear authors,**

**I congratulate you for this paper. The statistical analysis is robust; however, the manuscript will benefit from major changes before publication.**

**The authors used retrospective data to evaluate the impact of early nutritional therapy and mortality. Also they analyzed the route of nutritional therapy.**

**First, I suggest an english review of the manuscript - it is understandable, but difficult to read.**

**An example in the background:**

**"Nutritional support is also associated with neurological prognosis and mortality of neurocritically ill patients with stroke or traumatic brain injury [6-8]. Early nutrition is especially important because it can affect neurological prognosis."**

**Associated with increased risk of mortality or a reduction in mortality? can affect neurological prognosis in what way - good or bad? And this same kind of sentence is repeated throughout the manuscript.**

**I will comment each part of the manuscript separately.**

R. We appreciate valuable comments. We have revised the redundant phrase. In our study, neurological outcomes were not investigated. Primary endpoint was in-hospital mortality. A more accurate expression is “EEN may reduce the risk of mortality in neurocritically ill patients”. We changed conclusion as “EEN may reduce the risk of in-hospital mortality and infectious complications in neurocritically ill patients.” In addition, as your recommendation, we revised each part of the manuscript separately.

**Abstract:**

**Background - there is no background information, only the objective of the study. Please include a sentence that explains the current knowledge.**

**Methods - no study design (retrospective cohort…)**

**Conclusion - can be clearer - again english editing.**

R. As your comments, we revised the manuscript. Revised sentences have been underlined and highlighted in the revised manuscript for your convenience.

**Background:**

**Better review of literature. Bring the hard data from previous study to support your argument (meaning mortality reduction in % (p=...) or no statistical difference in other study (p =...)). Make it clear what new information this study adds**

R. As your comment, we revised the section of Background. We provided accurate data and p-value in the text as the following sentences (line 35-42 in page 5).

Malnutrition is associated with poor clinical outcomes such as higher rates of mortality (32% vs. 14%, P=0.018) [Ref. 4], nosocomial infection (23.4% vs. 3.5%, P<0.001) [Ref. 5], and long stay in intensive care unit (ICU) [Ref. 4,6]. Similarly, in critically ill patients with stroke or traumatic brain injury, nutritional support is associated with neurological prognosis and mortality [Ref. 7-9]. In stroke patients, the mortality rate of malnourished patients was 37%, which was significantly higher than that of patients with normal nutrition which was 21% (P<0.001) [Ref. 9]. In traumatic brain injury, it was reported that the rate of infection was reduced in early nutrition compared to delayed nutrition (risk ratio: 0.77, P=0.04) [Ref. 8].

**Methods:**

**The methods and statistical analysis are clear**

R. We appreciated your kind comment. We performed various statistical methods to correct the bias. However, as other reviewer’s opinion, various statistical methods can confuse the readers. Therefore, PSTW (a relatively rare method) and IPTW (an inappropriate balance, frequently over 0.1 of SMD) has been removed from the original manuscript.

**Result:**

**The results are confusing.**

**I suggest dividing the results into the outcomes: primary (mortality) and secondary (infections complications) and not according statistical analysis.**

R. As your comment, we revised the section of Results (line 138-171).

**Also, table 2 - the p is for the comparasion between the 3 groups (late nutrition, EPN and EEN) or between (non-EEN and EEN). It is not clear.**

R. We apologize for the lack of clear statement. We compared late nutrition, EPN, and EEN. We added the following legend indicated what comparison to revised table 2.

Data show a comparison between late nutrition, EPN and EEN. (the legend of revised Table 2)

As other reviewer’s recommendation, we re-analyzed the difference between EEN and non-EEN (only late nutrition, except EPN). Late parenteral nutrition may be associated with faster recovery and fewer complications, as compared with EPN (Ref. 21). Therefore, combining the EEN and the EPN groups, or the late nutrition and the EPN group might not be useful to evaluate the association between nutrition types and clinical outcomes. EPN might affect the outcome as a confounding factor. We revised Figure 2 & 3.

Ref. 21: Casaer MP, Mesotten D, Hermans G, Wouters PJ, Schetz M, Meyfroidt G, et al. Early versus late parenteral nutrition in critically ill adults. N Engl J Med. 2011; 365: 506-17. https://doi.org/10.1056/NEJMoa1102662 PMID: 21714640

**5.Discussion:**

**The comparison between current literature and the study result has room to be improved, but mostly English editing will help a lot.**

**There was a lot a of EPN. Any reason why? It is not supported by any guidelines. This should be better addressed in the discussion.**

**Do you have data regarding calories and protein intake in each group? I imagine that those in the early group would have a lesser caloric and protein deficit**

R. We agree with the reviewer's comments. In the early stages, the sympathetic tone may be elevated in patients with elevated intracranial pressure (Ref. 8). Therefore, paralytic ileus, abdominal distension, and vomiting may not be uncommon in these patients. Hence, PN would be preferred occasionally compared with EN in our center.

There was the limitation of this study. Unfortunately, the amount of EEN or EPN calorie intake for patients in the early stage was not considered in this study due to its retrospective nature. We have mentioned these limitations in the limitation section of the revised manuscript.

The amount of EEN or EPN calorie intake for patients in the early stage was not considered in this study due to its retrospective nature.

Ref. 8: Wang X, Dong Y, Han X, Qi XQ, Huang CG, Hou LJ. Nutritional support for patients sustaining traumatic brain injury: a systematic review and meta-analysis of prospective studies. PLoS One. 2013; 8: e58838. https://doi.org/10.1371/journal.pone.0058838 PMID: 23527035

As your recommendation, our manuscript **has been proofread by a professional English editing service.** We thank the reviewer for valuable comments. Addressing them fully has significantly strengthened the manuscript.
