# Supplementary material for: Impact of early nutrition and feeding route on clinical outcomes of neurocritically ill patients
Source: PLoS One. 2023 Mar 23;18(3):e0283593. doi: 10.1371/journal.pone.0283593 (PMC10035931; doi:10.1371/journal.pone.0283593)
Supplement: Attachment — Submitted filename: Response to reviewers PLoS ONE_minor_20220902.docx [file pone.0283593.s004.docx]

September 2 2022

Dr. Rishabh Charan Choudhary

PLOS ONE

Manuscript ID: PONE-D-21-39517

Title: Impact of early nutrition and feeding route on clinical outcomes of neurocritically ill patients

Dear Dr. Rishabh Charan Choudhary

Thank you very much for your letter and for the helpful comment from the reviewer. We appreciate the opportunity to resubmit our revised manuscript entitled **“Impact of early nutrition and feeding route on clinical outcomes of neurocritically ill patients”**. As always, you and your editorial staff have again provided us with a comprehensive and prompt review. Many of the valuable and constructive points that the reviewers pointed out were well taken by all the authors. After going over the reviewer’s comments, my colleagues and I have performed additional investigation and made some revisions in hopes of improving our paper. The revised and added portions of the manuscript are stated in the “Response to Reviewers” and are underlined and highlighted in the revised manuscript for your convenience.

We hope that, upon review, our study will be found to be meritorious of publication in the PLOS ONE.

Yours sincerely,

Jeong-Am Ryu, M.D., Ph.D.

Department of Critical Care Medicine and Department of Neurosurgery, Samsung Medical Center, Sungkyunkwan University School of Medicine, 81 Irwon-ro, Gangnam-gu, Seoul 06351, Republic of Korea

Tel: 82-2-3410-6399, Fax: 82-2-2148-7088

E-mail: lamyud.ryu@samsung.com

**Response to Reviewers**

**Reviewer #1:**

**Reviewer #1: Second Review**

**Thank you for the revised manuscript. It is much improved.**

**Concerns:**

**The legend for Table 2 clarifies the comparison. However, since there are three groups being compared it would seem that comparison of continuous variables would have required ANOVA rather than Student’s t-test. If this was done, please add this to the description of the data analysis. If this was not done, please explain what method of analysis was used to compare data for continuous variables in three groups.**

**In Figure 2, there are lines for the IPTW analysis, but you reported removing this analysis from the manuscript. It should be removed from the Figure as well.**

**Finally, I previously recommended that you compare the quantity and the composition of the nutrition provided but you indicated that these data are not available. You also indicated that you mentioned this as a limitation in your discussion section. I would encourage you to expand upon this in the discussion. Because the data for the quantity and the composition of the nutrition provided were not available, you are only comparing the timing of initiating nutrition by either the enteral or the parenteral route. However, the quantity of nutrition may have an impact on the findings of your study. Some previous studies have shown a difference in outcomes based on calories delivered, based on protein delivered, and some on both calories and protein delivered. This is an unfortunate deficit in your study.**

R1. We apologize for the lack of clear statement. We compared late nutrition, EPN and EEN in original manuscript. In table 2, we used one-way analysis of variance (ANOVA) for continuous variables. We performed ANOVA analysis using the tableone package and the moonBook package of R program.

We added the following sentences in the Methods section of the revised manuscript

Line 90-92: Data were compared using Student’s *t*-test and one-way analysis of variance for continuous variables and Chi-square test or Fisher’s exact test for categorical variables.

R2. In revised figure 2, there was no line for IPTW. We showed the line for IPTW only in the first rebuttal letter for your comprehension.

R3. We agree with the reviewer’s comment. Unfortunately, the amount of EEN or EPN calorie intake for patients in the early stage was not considered in this study due to its retrospective nature. As your recommendation, we added these limitations in the discussion section of the revised manuscript.

Line 244-253: Adequate calorie and protein intake is important for recovery in critically ill patients [33,34]. Adequate nutritional support may also be important in neurocritically ill patients. The patients with severe traumatic brain injury have increased energy expenditure usually increase by 87%–200% above the usual requirement and may be elevated for 30 days due to metabolic changes [26,35,36]. In addition, systemic catabolic change could lead to hyperglycemia, protein wasting, and increased calorie demands [26,35]. Therefore, optimized calorie and protein supply is also important for acute brain injury patients. However, energy expenditure, calorie and protein intake were not considered in this study. Accurate analysis of calorie and protein supply and energy expenditure may be necessary to investigate the relationship between early nutrition and clinical outcomes in neurocritically ill patients.

Ref 26.	Ohbe H, Jo T, Matsui H, Fushimi K, Yasunaga H. Early enteral nutrition in patients with severe traumatic brain injury: a propensity score-matched analysis using a nationwide inpatient database in Japan. Am J Clin Nutr. 2020; 111: 378-84. https://doi.org/10.1093/ajcn/nqz290 PMID: 31751450

Ref 33. Yamamoto S, Allen K, Jones KR, Cohen SS, Reyes K, Huhmann MB. Meeting Calorie and Protein Needs in the Critical Care Unit: A Prospective Observational Pilot Study. Nutr Metab Insights. 2020; 13: 1178638820905992. https://doi.org/10.1177/1178638820905992 PMID: 32153344

Ref 34.	Hartl WH, Kopper P, Bender A, Scheipl F, Day AG, Elke G, et al. Protein intake and outcome of critically ill patients: analysis of a large international database using piece-wise exponential additive mixed models. Crit Care. 2022; 26: 7. https://doi.org/10.1186/s13054-021-03870-5 PMID: 35012618

Ref 35. Abdullah MI, Ahmad A, Syed Saadun Tarek Wafa SWW, Abdul Latif AZ, Mohd Yusoff NA, Jasmiad MK, et al. Determination of calorie and protein intake among acute and sub-acute traumatic brain injury patients. Chin J Traumatol. 2020; 23: 290-4. https://doi.org/10.1016/j.cjtee.2020.04.004 PMID: 32423779

Ref 36.	Kurtz P, Rocha EEM. Nutrition Therapy, Glucose Control, and Brain Metabolism in Traumatic Brain Injury: A Multimodal Monitoring Approach. Front Neurosci. 2020; 14: 190. https://doi.org/10.3389/fnins.2020.00190 PMID: 32265626

We thank the reviewer for valuable comments. Addressing them fully has significantly strengthened the manuscript.

**Reviewer #2:**

**Reviewer #2: Dear authors,**

**Thank you for your answers.**

**I do believe the manuscript is improved and all questions have been adequately answered.**

R. We thank the reviewer for valuable comments. Addressing them fully has significantly strengthened the manuscript.
